# When Essential Thrombocythemia Goes Triple-Negative: A Case of Acquired von Willebrand Disease

**DOI:** 10.7759/cureus.75265

**Published:** 2024-12-07

**Authors:** Sonal Kumar, Saad Sabbagh, Yehuda Galili, Steve Carlan

**Affiliations:** 1 Vascular Surgery, Ross University School of Medicine, Miramar, USA; 2 Hematology and Oncology, Cleveland Clinic Florida, Weston, USA; 3 Academic Affairs and Research, Orlando Regional Medical Center, Orlando, USA

**Keywords:** acquired von willebrand disease, bone marrow fibrosis, bone marrow morphology, calr mutation, essential thrombocythemia, jak2 v617f, mutation negative, myeloproliferative neoplasms, thrombosis risk, triple negative

## Abstract

Essential thrombocythemia (ET) is a type of myeloproliferative neoplasm (MPN) disorder characterized by persistent thrombocytosis and characterized by frequent association with cellular genetic alterations. The 10%-15% of ET that is not associated with genetic abnormalities is known as triple-negative essential thrombocythemia (TNET). A common complication observed in around 20% of ET patients is the development of acquired von Willebrand disease (AvWD). Acquired von Willebrand disease affects 10-20% of myeloproliferative neoplasm cases, often linked to essential thrombocythemia with extreme thrombocytosis, but its incidence in triple-negative ET is undiscovered. We present the case of a 43-year-old male who was referred to our clinic with a platelet count of 844 x 10^3^/µL and a workup including bone marrow aspirate and pertinent labs revealing TNET. A four-year history of platelet counts was available, revealing a gradually progressive trend upward. Acquired von Willebrand disease appeared approximately one month before his platelet count peaked at 1,200 x 10^3^/µL, and hydroxyurea and aspirin were started. The platelets gradually decreased and stabilized by six months around 400 x 10^3^/µL. The complexities of this case lie in the dual management of thrombocytosis-related thrombotic risks and AvWD-associated bleeding tendencies, exacerbated by the absence of standardized treatment guidelines tailored specifically to this patient subgroup. In this case, TNET and AvWD patients with a four-year survey of platelet counts demonstrated a good response to oral aspirin and oral hydrea daily.

## Introduction

Essential thrombocythemia (ET) is a myeloproliferative neoplasm (MPN) identified by persistent thrombocytosis and increased risk of thrombotic and hemorrhagic events. JAK2, CALR, and MPL mutations frequently accompany patients with ET; however, there is a small subset of patients that present without mutations and are identified as having triple-negative essential thrombocythemia (TNET) [1]. It has been found that between 10% and 15% of cases of ET are TNET and lack driver mutations [2]. Patients with mutation-negative ET can exhibit diverse clinical phenotypes and may harbor alternative genetic abnormalities contributing to their disease pathogenesis [3]. Due to the rarity of TNET, literature describing the clinical presentation and the management of patients with TNET is scarce. Previous research was inconsistent in revealing differences in survival outcomes from patients with driver mutations despite showing a lower incidence of thrombosis. Additionally, data on response to treatment and other complications are still lacking. A common complication observed in around 20% of patients with ET is the development of acquired von Willebrand disease (AvWD) [4]. Acquired von Willebrand disease is characterized by a qualitative or quantitative deficiency of von Willebrand factor (vWF) and can exacerbate bleeding symptoms in patients with underlying thrombocytosis [5], posing both diagnostic and therapeutic challenges. Three-quarters of patients with AvWD had platelet counts between 450,000 and one million, indicating that the index for suspicion is higher in patients with high platelet counts [5]. Although AvWD frequently presents in ET patients with high platelet counts, it has rarely been described in TNET patients, which adds to the complexity of management. Here we present the case of TNET complicated by AvWD in an asymptomatic 43-year-old male with hyperlipidemia. We discuss the clinical presentation, diagnostic evaluation, and management strategies employed in this rare case. Additionally, we review the relevant literature regarding the association between TNET and AvWD, emphasizing the importance of recognizing and managing this rare hematological coagulopathy.

## Case presentation

A 43-year-old male with a medical history of hyperlipidemia presented to the hematology specialty clinic for an evaluation of thrombocytosis. The patient complained of early satiety but denied other symptoms. The patient denied abdominal pain, headache, chest, or lower extremity pain. His personal and family history was negative for cancer, bleeding, or clotting disorders. Vital signs were within normal limits. The physical exam was unremarkable for lymphadenopathy, splenomegaly, or hepatomegaly. 

The differential diagnosis included reactive versus primary thrombocytosis. Initial work-up was negative for driver mutations, including JAK2, CALR, MPL, and BCR-ABL alterations. Peripheral blood smear was unremarkable, revealed increased platelet count, and the platelets were normal in appearance. Furthermore, the complete metabolic panel, lactate dehydrogenase, haptoglobin, and Coombs tests were also negative. Further workup at subsequent clinical encounters was negative for C-reactive protein, erythrocyte sedimentation rate, rheumatoid factor, and antinuclear antibodies. The patient was evaluated by the infectious diseases team, and the workup was negative for HIV, rapid plasma reagin (RPR), and hepatitis panel. Abdominal ultrasound showed normal spleen size.

**Figure 1:**
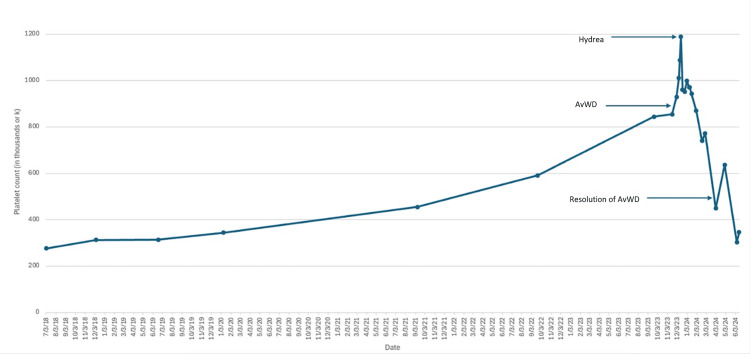
The relationship between the patient's platelet counts and key clinical events over time hydrea: hydroxyurea; AvWD: acquired von Willebrand disease

On initial presentation, the patient appeared well-nourished, in no acute distress, and laboratory findings were significant for a platelet count of 844 x 103/µL (150-450 x 103/µL), a white blood cell count of 7.2 x 103/µL (4.5-11 x 103/µL), hemoglobin 15.8 g/dL (13.5-17.5 g/dL), mean corpuscular volume (MCV) 89.3 fL (femtoliters, 80-100 fL), and a hematocrit 47.7% (41%-50%). Of note, the platelet trends over the previous four years have shown a gradually progressive trend upwards (Figure 1).

Initially, from 2018 through mid-2020, platelet counts remained stable at around 300,000 cells/μL. However, starting in mid-2020, there was a gradual increase, reaching approximately 600,000 cells/μL by late 2022, suggesting an underlying condition influencing platelet production. In early 2024, with further platelet elevation, AvWD appeared (indicated by the arrow in Figure 1), possibly secondary to the high platelet levels, which can disrupt vWF functionality. When platelet counts exceed 1,200,000 cells/μL, hydroxyurea treatment (indicated by the arrow in Figure 1) was initiated to manage the elevated levels. The response to hydroxyurea was notable, with a rapid decline in platelet count, indicating effective treatment. As platelet counts normalized, there was a concurrent resolution of AvWD, suggesting that restored platelet levels helped alleviate the acquired bleeding disorder (indicated by the arrow in Figure 1). By mid-2024, platelet counts stabilized around 400,000 cells/μL, likely due to the continued treatment efficacy of hydroxyurea. This favorable response was a reaction to hydroxyurea and was characterized by a rapid decline in platelet count, indicating effective treatment. As platelet counts normalized, there was a concurrent resolution of AvWD, suggesting that restored platelet levels helped alleviate the acquired bleeding disorder (indicated by the arrow in Figure 1). The reactive response to hydroxyurea resulted in the patient’s platelet counts returning to baseline once the underlying cause was addressed with the hydroxyurea. Overall, this graph highlights the successful management of elevated platelet counts and associated AvWD, demonstrating the therapeutic impact of hydroxyurea in such cases.

Due to the negative lab results and the progressive increase in platelet count, a bone marrow biopsy and aspirate were performed. Bone marrow revealed normocellular (~60% cellular) bone marrow with megakaryocytic hyperplasia, atypia, and mild reticulin fibrosis. Cytogenetics were normal: 46, XY. Next-generation sequencing study for myelodysplastic syndrome/chronic myelomonocytic leukemia profile, as well as cytogenetic analysis, showed no abnormalities. 

Additionally, peripheral blood Neo Comprehensive™ - Myeloid Disorders assay (NeoGenomics Laboratories, Fort Myers, FL, USA) was negative for any mutations. A positron emission tomography (PET) scan and a computed tomography (CT) scan showed negative fluorodeoxyglucose (FDG) (not currently inflamed/infected) lesions. A colonoscopy and esophagogastroduodenoscopy (EGD) done at an outside facility was normal.

The patient was started on oral aspirin 81 mg daily. Once the platelet count reached over one million, the von Willebrand assay was checked to rule out acquired deficiency in the setting of extreme thrombocytosis. The functional activity of vWF was mildly decreased. Due to the concern for bleeding, we started the patient on hydroxyurea for cytoreduction to decrease platelet count. After a few weeks, the patient's platelet count normalized to the target goal between 100-400 x 103/µL. Repeat vWF assay six weeks after cytoreduction initiation revealed the resolution of AvWD. 

## Discussion

The diagnosis of TNET requires careful integration of clinical, laboratory, and genetic findings to distinguish it from other causes of thrombocytosis and establish appropriate management strategies. Few papers discuss ET cases that lack mutations in driver genes such as JAK2, CALR, and MPL, making management and initiating appropriate therapy for TNET particularly challenging [6]. It is important to note that the presentation of TNET can vary widely among patients, and some individuals may remain asymptomatic for extended periods, such as the patient presented in this case, with the condition only detected through routine blood tests. These cases also have overlapping symptoms of thrombocytosis-related platelet dysfunction and bleeding tendencies characteristic of AvWD, making therapy such as aspirin potentially contraindicated [7]. 

According to the World Health Organization (WHO) criteria for ET diagnosis, the patient must have all four of the following: sustained platelet counts >450 x 103/µL/L, a bone marrow biopsy showing an increased number of enlarged and mature megakaryocytes with no significant increase or left shift of granulopoiesis or erythropoiesis, the presence of driver mutations/clonal markers, or the absence of evidence of iron deficiency or cause for reactive thrombocytosis, and not meeting WHO criteria for polycythemia vera or other myeloid neoplasms [6]. The patient presented above met the criteria for ET with no driver mutations. The patient’s initial platelet count was 844 x 103/µL, and a bone marrow biopsy revealed normocellular bone marrow with megakaryocytic hyperplasia, atypia, and mild reticulin fibrosis. 

Treatment strategies for AvWD-ET patients lack consensus guidelines, necessitating individualized approaches that balance the risks of bleeding and thrombotic events (11). The treatment for TNET typically involves low-dose aspirin for thrombosis prevention and may include cytoreductive therapy such as hydroxyurea or interferon-alpha for high-risk patients with significant thrombotic or hemorrhagic complications [8]. Traditional therapies such as desmopressin and vWF replacement pose challenges in dosing and efficacy assessment [8]. The lack of standardized protocols highlights the need for multidisciplinary collaboration and additional research to address this clinical population effectively. Long-term management requires vigilant monitoring of bleeding symptoms, platelet counts, and vWF levels to tailor therapeutic strategies and mitigate complications effectively. Patient education plays a crucial role in enhancing treatment adherence and early recognition of bleeding or thrombotic events. 

The prognosis for TNET is generally favorable with a lower risk of thrombotic complications and a more indolent disease course compared to other forms of ET [7], but regular monitoring and adherence to treatment are essential for optimal management and outcomes. Patients may develop significant vascular complications, such as splenic infarction [6], in addition to fatal complications.

## Conclusions

We presented the case of an asymptomatic 43-year-old patient with triple-negative ET and AvWD who demonstrated a successful response to aspirin and hydroxyurea. We emphasized the importance of continuous management of thrombosis and bleeding risks, as effective care for this rare patient subtype relies on regular monitoring of bleeding symptoms, platelet counts, and vWF levels to prevent complications. We advocate for a collaborative, multidisciplinary approach to ensure individualized care, detect potential issues early, and address the distinct challenges associated with this rare condition. 

## References

[1] Marzollo A, Zampieri S, Barozzi S (2024). Thrombocytopenia 4 (THC4): six novel families with mutations of the cytochrome c gene. Br J Haematol.

[2] Milosevic Feenstra JD, Nivarthi H, Gisslinger H (2016). Whole-exome sequencing identifies novel MPL and JAK2 mutations in triple-negative myeloproliferative neoplasms. Blood.

[3] Langer AL, Connell NT (2021). Acquired von Willebrand syndrome. Hematol Oncol Clin North Am.

[4] Mital A, Prejzner W, Bieniaszewska M, Hellmann A (2015). Prevalence of acquired von Willebrand syndrome during essential thrombocythemia: a retrospective analysis of 170 consecutive patients. Pol Arch Med Wewn.

[5] Kanderi T, Puthenpura M, Shrimanker I, Sapna F, Felter SC (2020). Triple-negative essential thrombocythemia complicated by thrombosis and acquired von Willebrand disease in a young man. Am J Case Rep.

[6] Ozogbo S, Kim J, Olafimihan A (2023). A rare case of triple negative essential thrombocythemia presenting with splenic infarction: a diagnostic conundrum. Blood.

[7] Cattaneo D, Croci GA, Bucelli C (2021). Triple-negative essential thrombocythemia: clinical-pathological and molecular features. A single-center cohort study. Front Oncol.

[8] Cattaneo M (2023). Aspirin in essential thrombocythemia. For whom? What formulation? What regimen?. Haematologica.

